# Supplementary material for: A 3-D groundwater isoscape of the contiguous USA for forensic and water resource science
Source: PLoS One. 2022 Jan 7;17(1):e0261651. doi: 10.1371/journal.pone.0261651 (PMC8741010; doi:10.1371/journal.pone.0261651)
Supplement: S1 Fig — White = no wells at any depth; Grey = no wells at current depth; Blue = only well completion database wells at current depth; Red = only isotope database wells at current depth; Purple = wells from both databases at current depth. Z index represents the subsurface depth layer, where: 1 = 500–2,000 m; 2 = 200–500 m; 3 = 100–200 m; 4 = 50–100 m; 5 = 25–50 m; 6 = 10–25 m; 7 = 1–10 m. Views can be loaded as a widget in most web browsers, and users can move through the X/Y/Z slices using the arrow and page up/down keys. The 3-d model can be rotated by clicking and dragging the view frame. (HTML) [file pone.0261651.s001.html]

cubeView
