# Supplementary figures and images for: A 3-D groundwater isoscape of the contiguous USA for forensic and water resource science
Source: PLoS One. 2022 Jan 7;17(1):e0261651. doi: 10.1371/journal.pone.0261651 (PMC8741010; doi:10.1371/journal.pone.0261651)

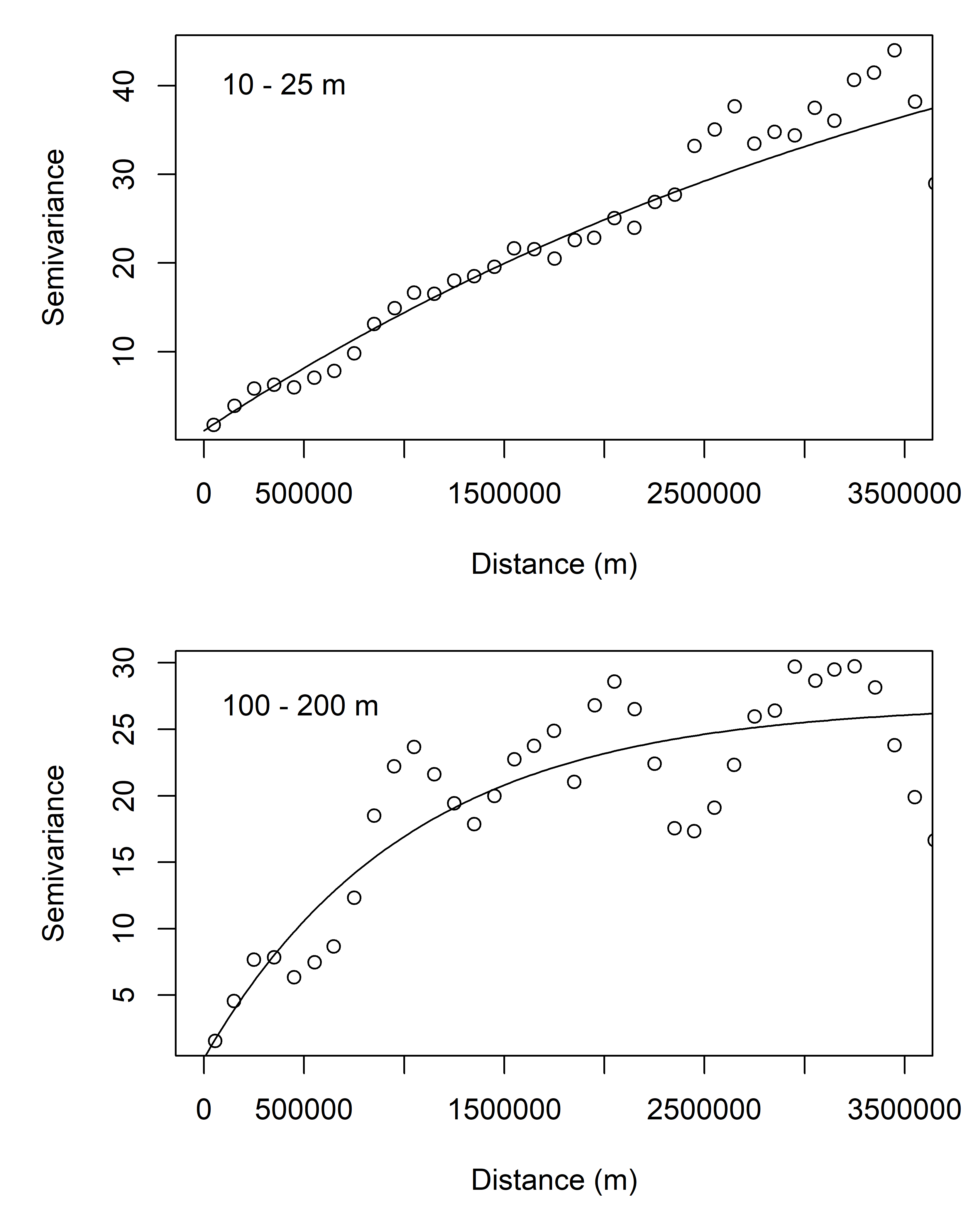

Supplement: S2 Fig — (PNG) [file pone.0261651.s002.png]

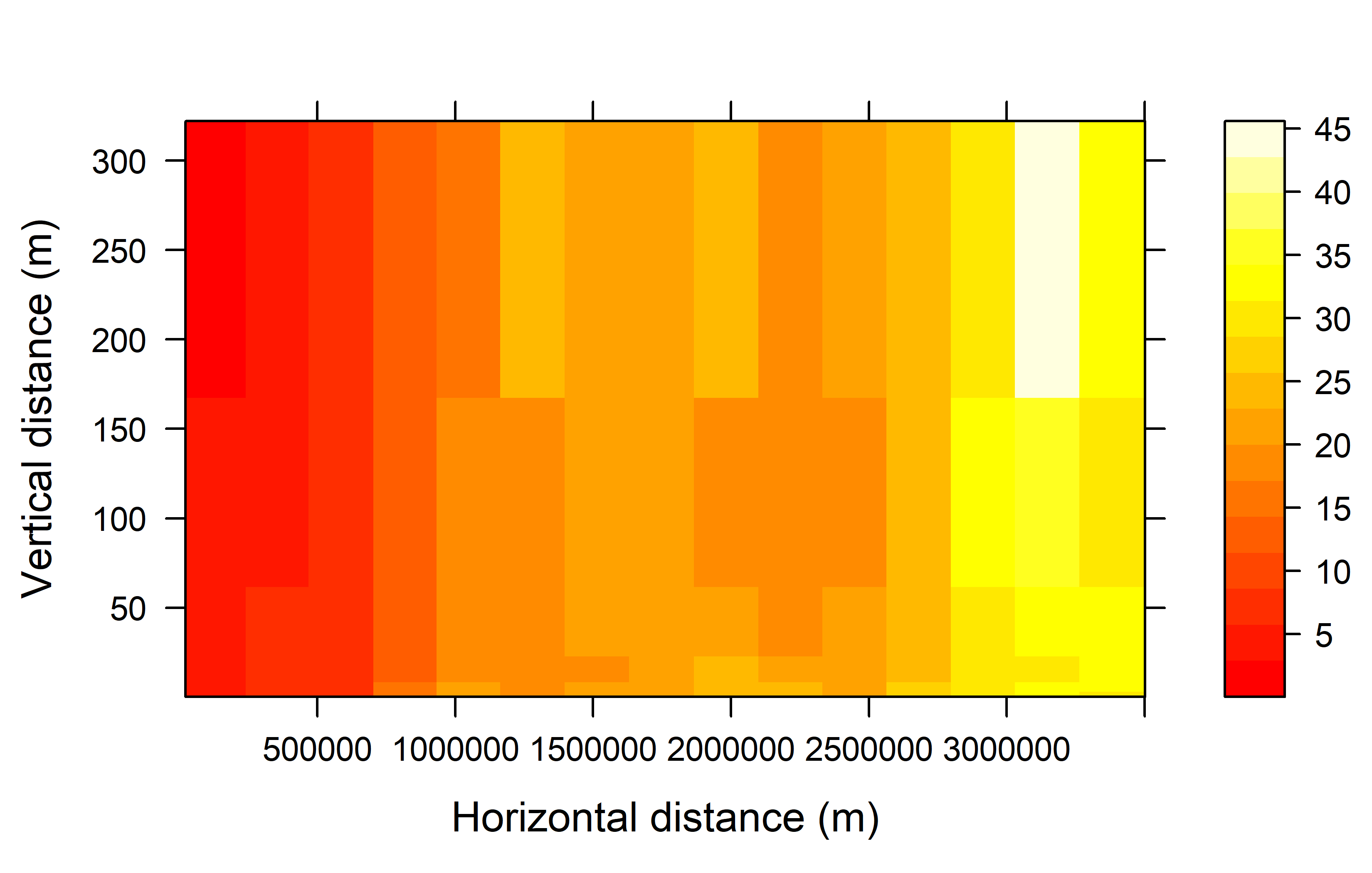

Supplement: S3 Fig — (PNG) [file pone.0261651.s003.png]

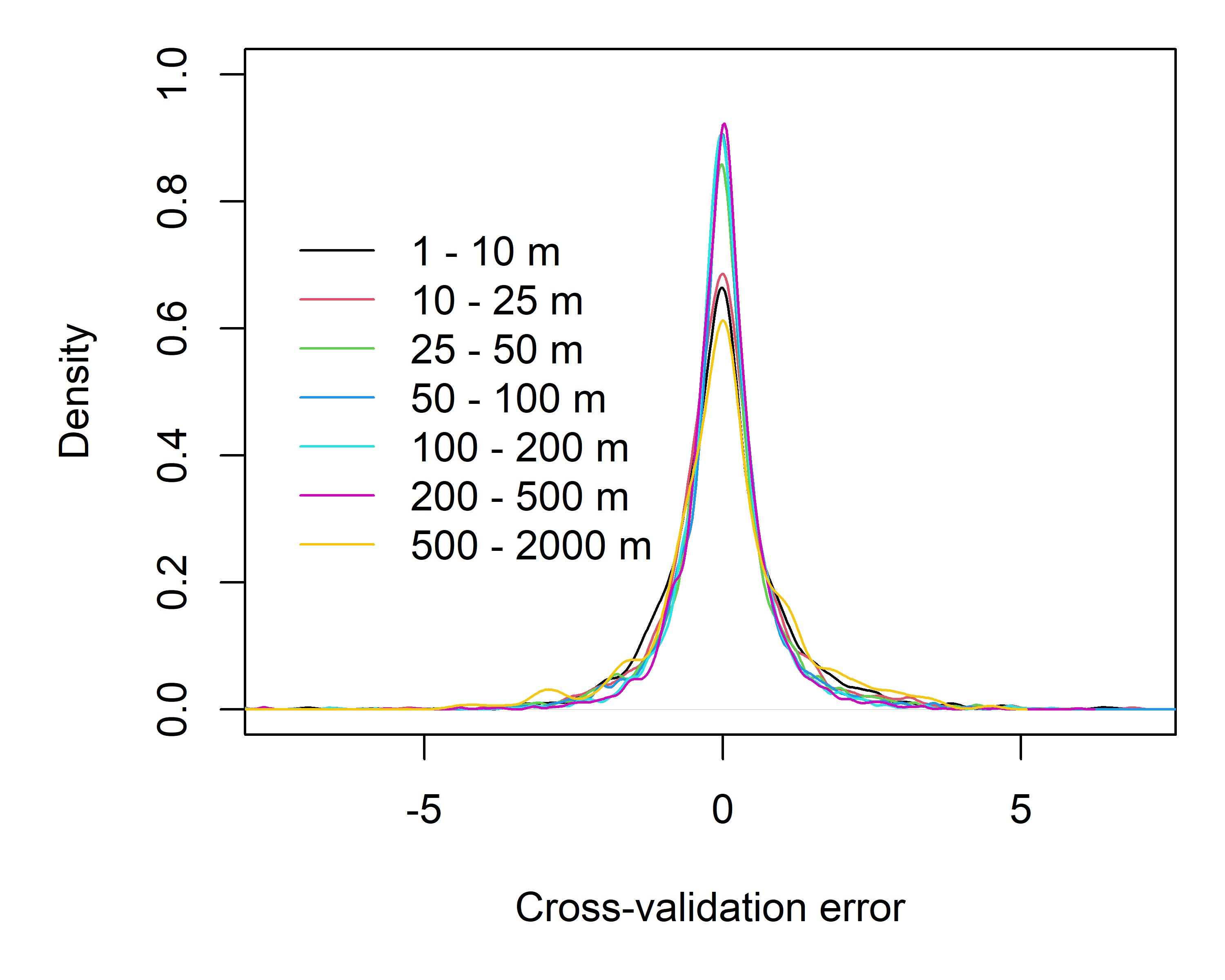

Supplement: S4 Fig — (PNG) [file pone.0261651.s004.png]
