# Supplementary material for: A 3-D groundwater isoscape of the contiguous USA for forensic and water resource science
Source: PLoS One. 2022 Jan 7;17(1):e0261651. doi: 10.1371/journal.pone.0261651 (PMC8741010; doi:10.1371/journal.pone.0261651)
Supplement: Attachment — Submitted filename: 211005_response.docx [file pone.0261651.s010.docx]

Editor: In lieu of the comments provided by 3 reviewers, I recommend the paper has potential for publication in PLOS ONE with moderate revisions. In particular, I recommend the authors pay close attention on providing specifics and justifications where its been requested by the reviewers. Please go through the itemized list of comments that have been provided by the reviewers to amend your paper, and provide a response as you deem appropriate. Reviewer 2 has recommended major revisions, and I expect the authors will work on those in addition to some minor clarifications that have been suggested.

**We have thoroughly addressed all reviewer comments, and in almost all cases made additions or minor modifications to the manuscript and/or analyses in response to these. Below we note several places where reviewer 2 appears to have mis-read statements or mis-interpreted information or intentions in our original submission, and this is reflected in their comments. In most of these cases our response has included revision or addition of text to further enhance the clarity of our presentation and (hopefully) reduce the potential for mis-interpretation. In other cases this reviewer has made excellent suggestions for clarifying or further documenting our analyses, and we have generally adopted these suggestions. Detailed responses to all reviewer comments can be found below.**

Reviewer #1: This is a great paper and one that is of high value. The authors should be commended for their work. And as a reviewer, thanks for taking the time writing this to a high quality prior to submitting. I have a few minor things that should be addressed prior to publication:

1. Maps are static snapshots of data – but we know water is very dynamic, especially in the upper layers. The authors discuss the relationship between precip and groundwater in the upper layers and do make mention to the stability of their estimates, but do not fully discuss how these maps may change through time. Can we expect the upper layer maps to be static in ways similar to the lower layers?

**This is a great question. The dogma is that the isotopic composition of most groundwater systems is relatively static, but for shallow aquifers with very short transit times and/or systems that are being depleted through extraction this may not be the case. It is important for readers to understand the potential implications of such dynamic changes on the use of this product, so we have added several lines of text that address the issue. First, in the introduction:**

**“The temporal coverage of each [well record] data source also varied, though in general the digital data we obtained was biased toward wells completed in the past several decades. Because date of well completion was not always reported we did not systematically evaluate difference in the temporal coverage of different data sources.” and “The isotopic data represent samples collected between 1970 and present, with relatively constant temporal sample density between 1984 and 2019 and fewer samples in the earlier and most recent parts of the sampling interval.”**

**Second, in the discussion of the limitations of the data product (Conclusions section):**

**“…the isoscape is based on data (both well records and isotopic samples) that are aggregated over time and may be heterogeneously sampled in time. The product is thus best considered a climatological representation of recent water isotope patterns, but with the caveat that the results may be biased toward different parts of the observational period in different locations and may not reflect changes in dynamic groundwater systems, especially in very shallow aquifers and those which are being unsustainably extracted.”**

2. The maps are missing scales and N arrows. Outlines of aquifer regions can be better displayed.

**We have added a scale to each map. Because of the projection used the page direction of north changes across the map, so it doesn’t make sense to include a N arrow on our figures. The political boundary of the USA (and the individual states) is included on all figures and should provide concrete (and to most readers recognizable) frame of geographic reference for the maps. We have also revised the colors on Figure 2 in order to make the aquifer outlines more visible.**

3. I am curious why 25km cells were used to visualize the maps. With so many data points, it seems a smaller cell size would be possible. Was this done to minimize the gray area (i.e., places with no/missing data)?

**Reviewer 2 raised a similar point, and we provide a detailed response below.**

4. These types of big data papers are becoming more common but are still new to many readers – so having better documentation on the actual technology used in the data processing will help improve this new field, as well as acclimate others to the tools that are out there. You mention R packages, but in your acknowledgements, you also talk about high performance computing and data storage. Based on the data in this study, I am assuming there are other software programs used to help manage the data, as well as GIS to visualize the data. What were the technical specs on the computer used in this analysis (e.g., RAM). This should all be included in the methods, and will help statements this like “Because the computation time required to generate 3-d semivariograms from the full dataset would have been prohibitive” make sense.

**Thank you for these suggestions. In the methods section we now include the specs of the desktop PC used for the work, as well as the file size of the large well depth database, for reference, and state specifically that all data analysis was done in R. We have also reported the amount of time required to run the 3-d variogram analysis on the 4,000-sample subsets, for reference.**

Reviewer #2: This study tried to give a picture of the national-scale groundwater resources by compiling and analyzing existing groundwater data. Some of the findings are interesting, but many parts of the method section need much more and better descriptions and justification. Many parts of the result section read like pure speculations rather than being supported by the results. The goal of this study is still not clear, and it would be great if the authors can provide examples of showing how the analysis results and findings can help understand groundwater systems in practice. The descriptions and interpretation of the results are a bit scattered; I think this study has a couple of interesting and meaningful findings that the authors want to highlight and further describe in the manuscript. I think splitting the result section into result and discussion sections would help improve the flow and readability. Finally, the authors may want to select a couple of states to explain and interpret the results in detail. Here are my specific comments.

**Based on these (and other) comments, it seems that the reviewer has the impression that the goal of our work is to advance understanding of groundwater systems (recharge, flow, etc.). This is not the case – our study’s primary goal is stated in the final paragraph of the introduction: “Here, we attempt to advance the development of accurate predictive groundwater…”. This is primarily a data product development/methodological study, and our discussion is structured in accordance with this goal. In general, when we talk about hydrological processes, it is in the context of evaluating our results and data products relative to known hydrological processes to ‘reality check’ and/or highlight features of the results, and the progression is structured around the analysis steps and products rather than the processes or regions. After re-reading the manuscript, and given the positive reaction of the other two reviewers, we maintain that it is the best structure given the goals and focus of the paper.**

Line 61: Is this the same as what mentioned in Line 46? This looks like important, as it is one of the sources of data used in this study. Please provide a description about the CONUS somewhere in the manuscript.

**Line 61 (original ms line numbers) is in the final paragraph of the introduction, and states that our work begins with the development of a 3-d model for aquifer presence. Line 46 is in a paragraph describing previous work and references a study that used machine learning to model shallow groundwater isotope ratios. The two statements are unrelated except that the term CONUS appears in both (in both the previous study and ours this is the spatial domain under consideration).**

**We have reviewed the relevant text and believe that the meaning is clear, including the distinction between the prior study and the introduction of our own work, given the context in which these statements appear. The term CONUS is defined on line 46. The datasets used in our work are described in the subsequent section (Materials and Methods).**

Lines 71 to 72: Please describe the “state-level geodatabases” and “U.S. Geological Survey where state-agency data were unavailable” in terms of the data spatial coverage and temporal/spatial resolutions. I wonder if the spatial/temporal resolutions of the data compiled from the different sources (or states) are consistent with each other. If not, please explain who the inconsistency was considered in the analysis and prediction.

**We have added the following text to expand and elaborate, as requested:**

**“Each source consisted of georeferenced, tabular data records, usually reporting information on the location, depth, use, and date of completion of groundwater wells. The precision of geographic coordinate information varied among sources, and in some cases had been degraded (e.g., to the center of a State Plane Coordinate System section) prior to reporting. In all cases the precision of reported coordinates was far greater than the spatial resolution of our analysis, however. The temporal coverage of each data source also varied, though in general the digital data we obtained was biased toward wells completed in the past several decades. Because date of well completion was not always reported we did not systematically evaluate difference in the temporal coverage of different data sources.”**

Line 82: Is the number of samples, 210, large enough for this national scale analysis? Please justify the selection of the samples.

**These data represent a small fraction of the total dataset. We have added a statement here that clarifies that these data were collected to fill “identified gaps in the compiled dataset”.**

Lines 92 to 93: Please describe the sources of the information about the origin of tap water.

**This information is provided in the subsequent sentence: “…public water records (in most cases information reported in annual Consumer Confidence Reports, CCRs) or interview with owners or managers”.**

Line 100: Please provide an overview of the well and groundwater isotope databases used to develop a gridded, 3-d map of aquifer in this study, in terms of the quantity and quality.

**We are confused by this request, since the subsection “Materials and Methods, Data” which immediately proceeds the text referenced by the reviewer provides the requested information. We assume there’s just some confusion about the fact that the data sources described in that section are in fact those which are used in the aquifer mapping and have attempted to clarify this by editing the referenced text to read “We used the well and groundwater isotope databases described in the previous section…”.**

Line 108: Please justify the selection the spatial resolution, 25 km X 25 km, of a regular grid used in this study (rather than others such as 10 km X 10 km or 100 km X 100 km). I believe the cell size or spatial resolutions are critical to this study, as the determine the accuracy as well as precision of the final results or prediction. Please discuss the sensitivity of the analysis or prediction results to the cell sizes somewhere in the manuscript.

**Reviewers 1 and 2 made this point, and we acknowledge that the original manuscript didn’t adequately justify the choice of resolution. The selected resolution reflects a compromise. We would of course like to achieve the highest resolution possible. However, given that both the well completion and isotopic databases are incomplete and imperfect, this resolution was selected to develop a data product that allows us to represent 1) whether groundwater (from a given depth) is *likely to be available* to individuals at a given site and 2) its likely isotopic composition. Because we don’t expect a well record to be present at each location where water is available, we have chosen to generalize at a scale that we think is appropriate to the scale of most aquifers (and also allows that, in some cases, water may be laterally transferred some distance). We also note that the vast majority of the isotopic variation in the dataset occurs over length scales longer than 25 km, so we are not losing much precision by generating our data product at this scale. We have added the following to the methods section to provide justification for the selected resolution:**

**“This resolution was selected as a compromise based on practical (e.g., computational demand and data size, scaling of isotopic variance with distance) and theoretical (e.g., approximate length scale of aquifers, likely increase in mixing with depth) considerations. With respect to aquifer presence/absence, the 25 km spatial resolution is likely too coarse to represent some shallow, heterogeneous (e.g., alluvial) aquifers, but should be adequate to capture patterns of presence/absence of such aquifers and to map the general footprint of most basinal and regional aquifers. With respect to isotopic variability, the average variance among δ^18^O values from multiple wells within a voxel increases continuously with grid cell size, but remains below 0.53‰, similar to the uncertainty in the predicted mean grid cell values, for the 25 km grid (see below). Finally, our semivariogram analysis (see below) suggests that systematic spatial changes in groundwater δ^18^O values over 25 km are generally trivial (up to a few tenths of a per mil), implying this scale is adequate to resolve spatial patterns in groundwater isotopic composition.”**

Lines 117 to 118: Please provide technical details of the variogram analysis such as parameter values used in the spatial interpolation (or extrapolation).

**All semivariagram parameters appear in Table 1 in the manuscript and are discussed in the Results/Discussion section.**

Lines 124 to 126: It is still not clear why independent semivariogram modeling and kriging were used in this study. If the 3-d variograms showed systematic autocorrelation, I wonder what alternative analysis methods would be used.

**The text referenced here states that “…the 3-d variograms showed no systematic autocorrelation of isotope δ values in the depth dimension…”, and subsequent text in the Results/Discussion section goes on to discuss this result in more detail. Based on the reviewer’s comment is seems they may have mis-read this statement. The lack of autocorrelation in the depth domain reported here provides justification for proceeding with the 2-d modeling.**

Lines 131 to 134: The consistency of the isotope measurements would not be maintained by the averaging processes taken by the layers. I am not sure the “independence” assumption would be valid for the analysis.

**We aren’t sure what the reviewer means by “consistency” here. Averaging before interpolation was done primarily to increase computational efficiency – since we are conducting block kriging to predict the mean value within each grid cell, working with a dataset that represents observed cell means is reasonable and should not bias the result (although it may affect the smoothing of the final product to some degree). However, this is not strictly necessary, and to simplify our analysis and eliminate any potential confusion or skepticism that might arise from the use of cell averages we now use the individual data in making our kriging predictions. This actually improves our cross-validation results slightly (e.g., the highest RMSE for δ^18^O in a subsurface layer drops from 1.5 to 1.25‰), but otherwise has no concrete impact on the results or interpretations.**

**The comment on independence refers to our calculation of uncertainty for the geostatistical predictions. We calculate this using error propagation to combine uncertainties in the kriging prediction variance and the mean within-voxel variance calculated from the samples. We maintain that this is appropriate for the purposes of the analysis, given that our desire is to provide a quantitative estimate of the uncertainty that reflects the expected distribution of isotope values for individual wells within the voxel based on the predicted mean. We have expanded this text to further elaborate the justification for (and implications of) this approach:**

**“For the prediction uncertainty stack, values for each layer were calculated by propagating the block kriging prediction variance, which represents the uncertainty in the predicted mean value for that voxel, and the average within-voxel variance for that depth interval, representing the expected distribution of individual well values around the mean. Our propagation assumes independence of these two sources of uncertainty, which we believe is appropriate given that they represent two distinct error-generating processes, but to the degree that this assumption is incorrect the uncertainty estimates will be conservative.”**

Line 136: What does “modern” mean here?

**We have added “climatological, based on monitoring data from 1960 – present” to clarify this statement.**

Lines 136 to 137: I am not sure what “local groundwater – prediction isotope differences” mean or how they can be useful. In the result section, I found many discussions about this, but I am not sure the discussions are supported by the results.

**It appears that the reviewer has misread our text, which says “local groundwater – precipitation isotope differences” (not “prediction”). We can see how this misreading would lead to confusion. All we have done here is subtract the precipitation isoscape values from the co-located groundwater isoscape values. In order to try to make this more obvious and avoid any additional potential for confusion we have rewritten the statement to read “…to create maps of the difference between local groundwater and precipitation isotope predictions.”**

Lines 140 to 141: I am not sure the tap water samples can be a good dataset for the model validation, as tap water can travel longer than 2.5 km (the cell size or spatial resolution of the analysis or prediction) from its source (groundwater).

**The reviewer has misrepresented the grid cell size, which is 25 km. Regardless, it is possible that in some (likely rare) cases water may have been transferred from outside of the grid cell in which the tap water was collected, and we agree that it is worth considering the potential affect of this on the comparisons. Kriging is a smoothed interpolator, and reference to the maps in the manuscript shows that lateral variations in predicted values are generally small over scales of several grid cells (i.e., up to 100 or more km). We have added the following text to the referenced paragraph to justify why lateral transport is unlikely to significantly impact this analysis: “Although it is possible that water used at some of these sites may have been transported laterally (i.e., across grid cell boundaries) from its location of extraction, all source-water isoscapes used here are spatially smoothed and it is unlikely that lateral transport would lead to large or systematic inaccuracies in our validation analysis.”**

Lines 154 to 164: I believe this paragraph may belong to the method sand data section.

**We have reviewed the paragraph, which describes the properties of the compiled well completion database. Although we can see the reviewers point and this kind of information would appear in Methods in some papers, we maintain that it belongs in our Results section. In this case, the combined database is not a data source that we obtained from elsewhere, but rather one of the products of the work (compilation and integration of a large number of sources). Reporting the properties of the dataset here is thus akin to reporting summary statistics and other properties of a new dataset generated in a lab, which we would normally expect to see early in a Results section.**

Lines 191 to 193: Please clarify how such a dataset (or “product”) can be useful in practice.

**The reviewer is asking how the gridded aquifer map could be useful in practice. Our initial response to this question is to point out that it is the foundation of our paper and all the analyses within it, and that our introduction makes the case for why such a product is needed: part of predicting the isotope ratios of groundwater available at a site is predicting which groundwater resources are available at that site. Nonetheless, this comment suggests we may need to better emphasize the potential uses of this data product. The referenced lines are within a section focused on validation of the map, and we don’t think this is the right place for discussion of applications, so we have added the following to the conclusions section: “We suggest that the 3-d aquifer map presence/absence map may be useful in large-scale hydrology and water resource studies that require a spatially extensive (though low-resolution) model of aquifer distributions and/or models of what groundwater resources are actively exploited by humans”.**

Lines 189 to 213: Please describe the analysis results and compare them by states in a quantitative manner, such as using some descriptive statistics.

**We’re sorry, but we see no way to accomplish this. The reason that we are making the gridded subsurface aquifer map here is that no such data product exists at a scale relevant to our work (to our knowledge). Many individual aquifers have been modeled in at much finer scales, usually for groundwater flow modeling applications. Most frequently these models are in 2-d or do not reflect variation in aquifer thickness. In general, the 3-d models we have found are either restricted-access (e.g.,** [**http://northplainsgcd.org/aquifer-data-maps/**](http://northplainsgcd.org/aquifer-data-maps/)**) or targeted at representation of subsurface hydrogeological complexity at a scale and level of detail that makes them difficult to compare directly with our coarse-resolution aquifer presence/absence map (e.g.,** [**https://doi.org/10.5066/F7JM27T6**](https://doi.org/10.5066/F7JM27T6)**). Nor do they explicitly reflect which subsurface units are or are not being extracted at any given location (which is the basis for our model). At the national scale, the USGS Groundwater Atlas (which we reference throughout this section) is the most comprehensive data source that we know of but only provides quantitative information in 2-d (spatial extent of aquifers; only generalized depth information is reported). As a result, our approach in this section is to conduct a ‘reality check’ on our new national-scale aquifer model through qualitative comparison with the Groundwater Atlas.**

Lines 220 to 225: These finds are interesting. Please further describe these using plots that can show the close associations between them.

**We’re happy that the reviewer finds this association between the range of spatial autocorrelation in groundwater and precipitation isotopes to be interesting, indeed we think it’s a useful result that demonstrates the dominance of precipitation values as a control on spatial patterns in groundwater isotope ratios. We don’t believe it is a particularly surprising result, however (for example, we’ve found very similar associations in our previous work on continental-scale patterns in river water and tap water isotope ratios), and our judgement is that it’s not worthy of additional figures. We note that supporting information and figures are already present in the manuscript. Table 1 reports the semivariogram model ranges for each subsurface layer, and these can be compared directly with values reported for other water resources (like precipitation) in the literature. Figure S2 illustrates example semivariogram models for two depth layers, further documenting the length scale and pattern of correlation observed in the groundwater data set.**

Lines 225 to 229: These are also interesting, but the authors may want to cite studies that support these finding. In addition, I think the analysis methods used in this study might have affect the results.

**The reviewer doesn’t state which part of our analytical workflow they think might have affected the results or how, so it’s difficult for us to respond to this comment. Our finding here is simply that there is no strong, consistent autocorrelation of groundwater isotope values in the depth dimension across the USA, which is a direct result from the 3-d semivariogram modeling described earlier in the manuscript. We have added a citation to a recent paper showing strong heterogeneity in the vertical profile of groundwater ages across the northern USA (Starn et al 2021) to back up our suggested explanation for this result, as suggested.**

Reviewer #3: The authors present a new approach to model 3-D groundwater isoscapes on a large scale with an impressive compilation of groundwater isotope data across the United States. They applied both 2-dimensional and 3-dimensional variogram analysis and kriging to generate the first 3-D groundwater isoscape. The paper is well-written with appropriate literature background and context. The figures are neat and informative. I’m confident that the manuscript, the isotopic data, and the 3-D isoscape products will be useful to scientists interested in both basic areas such as hydrology and ecology, but also applied areas such as forensic sciences. The authors did an excellent job explaining possible drivers of groundwater's δ18O spatial pattern, both in-depth and in 2 dimensions, as well as demonstrating the relationship of groundwater's δ18O to that of meteoric and tap water.

I believe the paper would benefit from a process-based model involving empirical relationships with the possible drivers of the spatial and depth pattern, especially considering the need to extrapolate the information to regions with few samples. However, it is worth noting that the authors incorporated the complexity of 3-D groundwater stable isotope mapping and opened up a whole new range of possibilities for refining and using the isoscapes presented in the paper. Additionally, the authors are aware and pointed out the models' limitations, indicating ways to overcome these limitations in future works. With all that said, I do recommend this paper for publication in Plos One.

**We really appreciate the reviewer’s positive comments as well as their nudge toward process-based modeling. Although we are big fans of bringing process and first principles into models, in general, we think that this is beyond the scope of this manuscript given the scale at which we are working (and the additional data requirements and complexities of parameterization that would be associated). We see this as a great direction for future work and note that the reviewer’s comments here circle back around to the value of the current approach (even if it leaves additional opportunities ‘on the table’).**

Minor comments:

Line 88: In δ = (Rsample – Rstandard) / Rstandard, it remained to show that the δ value is multiplied by a thousand (δ = (Rsample – Rstandard) / Rstandard * 1000).

**Although this has been the tradition for writing the delta definition in many papers, the current IUPAC guidelines for reporting of isotopic data emphasize that the per mil unit, in itself, already accounts for the expression of value as parts per thousand, and thus inclusion of “* 1000” in the delta definition is unnecessary and/or incorrect (Coplen 2011). We appreciate that the mix of conventions in use is a point of confusion within the community but are trying to abide by the formal recommendations since we see that as the best path toward consistency in future work.**

Fig 2: The polygons showing the extent of the major aquifers could be more evident. Some readers may have difficulty seeing it.

**We have attempted to improve the quality of this figure, as described in response to reviewer 1’s comments.**

Line 192: The word “that” is written twice.

**Fixed, thanks!**

Line 313 – 315: The authors say the error of precipitation isoscapes may be one of the four factors that can contribute to observed groundwater-precipitation offsets. I wondered what the behavior of observed groundwater values against the regionalized cluster-based water isotope prediction (RCWIP) might look like (Terzer et al. 2013), or even the more recent RCWIP2 (Terzer-Wassmuth et al. 2021).

**Good suggestion. We downloaded the RCWIP2 product and compared it with our own precipitation isoscape and the groundwater maps…there were minor regional differences but no impact on the pattern of groundwater-precipitation offsets. We added this text to the referenced section of the manuscript: “Replication of the groundwater-precipitation comparison using another recently published precipitation isoscape (Terzer‐Wassmuth et al 2021) yielded very similar offset patterns, suggesting that data limitations, more than interpolation methodology, might limit the accuracy of these products if and where errors in the precipitation isoscapes are responsible for observed groundwater-precipitation offsets.”**

Fig S1; S5; S6; S7: I cannot see the cubeView of the supplementary figures. I can open the figures, but only a gray cube with the 2-D map appears on the cube surface. If the intention was to show the data in 3-D, there had to be an error because I can't see it. If the intention was to show in 2-D over a 3-D cube, I suggest presenting it as a conventional map like the other figures in the paper.

**It sounds like these 3-d models, which are most easily viewed in a web browser, were probably loading correctly for the reviewer but they may not have figured out how to move the view through the depth and x/y dimension slices. We have updated the caption for the first cubeview to include: “Views can be loaded as a widget in most web browsers, and users can move through the X/Y/Z slices using the arrow and page up/down keys. The 3-d model can be rotated by clicking and dragging the view frame.”**

References

Coplen TB. 2011. Guidelines and recommended terms for expression of stable‐isotope‐ratio and gas‐ratio measurement results. *Rapid Communications in Mass Spectrometry* 25: 2538-60

Starn JJ, Kauffman LJ, Carlson CS, Reddy JE, Fienen MN. 2021. Three‐Dimensional Distribution of Groundwater Residence Time Metrics in the Glaciated United States Using Metamodels Trained on General Numerical Simulation Models. *Water Resources Research* 57

Terzer‐Wassmuth S, Wassenaar LI, Welker JM, Araguás‐Araguás LJ. 2021. Improved high‐resolution global and regionalized isoscapes of δ 18 O , δ 2 H. *Hydrological Processes* 35
