# Supplementary material for: A 3-D groundwater isoscape of the contiguous USA for forensic and water resource science
Source: PLoS One. 2022 Jan 7;17(1):e0261651. doi: 10.1371/journal.pone.0261651 (PMC8741010; doi:10.1371/journal.pone.0261651)
Supplement: Attachment — Submitted filename: 211130_response.docx [file pone.0261651.s011.docx]

Editor: Thank you for providing your revised manuscript. Based on the revisions you have provided we believe the manuscript is worthy of publication in PLOS ONE. However, a few minor comments will need to be clarified before it can be accepted. In particular Reviewer 2 has itemized 7 points that will need clarifications to be incorporated as you provide your revision.

**We have addressed each of these points, as described below.**

Reviewer #2:

1. “Line 61 (original ms line numbers) is in the final paragraph of the introduction, and states that our work begins with the development of a 3-d model for aquifer presence. Line 46 is in a paragraph describing previous work and references a study that used machine learning to model shallow groundwater isotope ratios. The two statements are unrelated except that the term CONUS appears in both (in both the previous study and ours this is the spatial domain under consideration). We have reviewed the relevant text and believe that the meaning is clear, including the distinction between the prior study and the introduction of our own work, given the context in which these statements appear. The term CONUS is defined on line 46. The datasets used in our work are described in the subsequent section (Materials and Methods).”:
The expression is still not clear if CONUS means “contiguous United States” or the name of a dataset used in the study of (28). I think the authors may want to separate (CONUS) from (28) or [28]; it may be helpful if the expression can be revised to “conterminous United States (or CONUS) [28].”

**Thank you for the clarification. We have revised the text to separate the abbreviation and the citation number, resolving the ambiguity noted by the reviewer: “…and a second (28) using machine learning methods to model shallow groundwaters of the contiguous United States (CONUS).”**

2. “We are confused by this request, since the subsection “Materials and Methods, Data” which immediately proceeds the text referenced by the reviewer provides the requested information. We assume there’s just some confusion about the fact that the data sources described in that section are in fact those which are used in the aquifer mapping and have attempted to clarify this by editing the referenced text to read “We used the well and groundwater isotope databases described in the previous section…”:
I am sorry that the authors were confused with this suggestion. What I would like to suggest was to provide “an overview” of the data used as clearly stated in the comment. I know the authors described the data in the previous section, but it was hard for me to clearly understand the overall picture of data used in terms of their quantity and quality, as many different types (well depths, isotope ratios, and tap water samples) of data that came from different sources (as describe in Tables S1 and S2) were used for different purposes (model development and validation). For example, it may be helpful to have a table classifying the datasets (with abbreviations or simple names) by purposes, sources, and types.

**We have added a table describing the basic properties of each of the 3 datasets used in the study. We now introduce and refer to these datasets using three letter acronyms throughout the text.**

3. “These data represent a small fraction of the total dataset. We have added a statement here that clarifies that these data were collected to fill “identified gaps in the compiled dataset”:
Please elaborate on the “identified gaps.” I also found that the spatial coverages of data used in this study were not clearly described in the manuscript; please try to map the coverages (using points and/or polygons) of the individual data.

**We have added map panels to figure 1 that now show the spatial coverage of each of the groundwater datasets. We have used different symbology for the compiled and newly produced groundwater isotopic data to address the reviewer’s request for more context on the targeted sampling.**

4. “The text referenced here states that “…the 3-d variograms showed no systematic autocorrelation of isotope δ values in the depth dimension…”, and subsequent text in the Results/Discussion section goes on to discuss this result in more detail. Based on the reviewer’s comment is seems they may have mis-read this statement. The lack of autocorrelation in the depth domain reported here provides justification for proceeding with the 2-d modeling.:
I believe I did read the statement correctly. My question was “if the 3-d variograms showed systematic autocorrelation, I wonder what alternative analysis methods would be used” as the authors understood. I was curious about alternative methods that the authors might use when they found there was systematic autocorrelation of isotope δ values. The authors did not know if 3-d variograms might show so systematic autocorrelation or strong systematic autocorrelation, and I was wondering what kinds of methods and approaches we might be able to use if there was STRONG systematic autocorrelation.

**Thank you for this clarification. We have updated the text to add the requested information: “We used the results of the variogram analysis to determine whether prediction via 3-dimensional kriging was warranted. Given that no systematic autocorrelation of isotope δ values in the depth dimension was found (see results), 3-d kriging was not supported and our subsequent analysis used independent semivariogram modeling and 2-d kriging of groundwater isotope data from each subsurface layer.”**

5. “All semivariagram parameters appear in Table 1 in the manuscript and are discussed in the Results/Discussion section.”:
Please mention Table 1 at the end of this description.

**The fitted parameters are a result of the analyses performed in this study, and we don’t think it’s appropriate to reference them in the methods section since they are not discussed there.**

6. Regarding the first paragraph of the “Results and Discussion” section:
It must be up to the authors, but I still believe this belong to the method and data section, as this is a summary of raw data compiled from the sources (rather than being analyzed or processed).

**We appreciate the reviewer’s perspective but have already elaborated why we disagree and believe that the placement of this text is appropriate (copied below from our original response document). Given that 2 other reviewers read the manuscript and did not express any concerns related to this issue we think we are justified in leaving the content of this section as is.**

**“We have reviewed the paragraph, which describes the properties of the compiled well completion database. Although we can see the reviewers point and this kind of information would appear in Methods in some papers, we maintain that it belongs in our Results section. In this case, the combined database is not a data source that we obtained from elsewhere, but rather one of the products of the work (compilation and integration of a large number of sources). Reporting the properties of the dataset here is thus akin to reporting summary statistics and other properties of a new dataset generated in a lab, which we would normally expect to see early in a Results section.”**

7. Regarding the relationship between groundwater and precipitation isotopes:
I believe a plot similar to Figure 6 (of the original manuscript) can be helpful.

**We have produced the suggested plot and added it to the manuscript (now figure 4).**
